# Forecasting Events in the Complex Dynamics of a Semiconductor Laser with Optical Feedback

**DOI:** 10.1038/s41598-018-29110-5

**Published:** 2018-07-16

**Authors:** Meritxell Colet, Andrés Aragoneses

**Affiliations:** 1 Carleton College, Department of Physics and Astronomy, Northfield, MN 55057 USA; 2 Present Address: Department of Physics, Eastern Washington University, Cheney, WA 99004 USA

## Abstract

Complex systems performing spiking dynamics are widespread in Nature. They cover from earthquakes, to neurons, variable stars, social networks, or stock markets. Understanding and characterizing their dynamics is relevant in order to detect transitions, or to predict unwanted extreme events. Here we study, under an ordinal patterns analysis, the output intensity of a semiconductor laser with feedback in a regime where it develops a complex spiking behavior. We unveil that, in the transitions towards and from the spiking regime, the complex dynamics presents two competing behaviors that can be distinguished with a thresholding method. Then we use time and intensity correlations to forecast different types of events, and transitions in the dynamics of the system.

## Introduction

Nature presents many physical systems where the interplay between a deterministic behavior, stochasticity and time delay leads to a broad variety of complex dynamics^1–3^. These complex systems, constituted by numerous elements interacting non-linearly, present collective emergent phenomena that can not be explained by analyzing its elements individually, but a broader approach is necessary to unveil any hidden structure in its dynamics. Some complex systems manifest their emergent behavior through sequences of extreme oscillations or spiking events. This type of behavior can be found in earthquake activity^4–6^, neuronal dynamics^7,8^, social networks^9^, heartbeat behavior^10–12^, optical systems^13^, stock markets^12,14^, among others^15–17^.

Understanding and characterizing the different dynamic regimes that a given system can manifest is relevant to forecast unwanted extreme events, or to distinguish between two competing behaviors that can lead to undesired events^4,18–20^.

Semiconductor lasers with optical feedback have shown to manifest a wide range of complex dynamics, from periodicity to high dimensional chaos^13^. Control and entrainment of these dynamics has practical applications, from encrypted telecommunications^21^, or subwavelength position sensing^22^, to reservoir computing^23^. One particular complex dynamics that semiconductor lasers with feedback can exhibit is the Low Frequency Fluctuations (LFF)^24^. In this regime the laser presents an excitable behavior^25^, i.e., for perturbations below a threshold the response of the system is linear and of small magnitude, but for perturbations above the threshold the response drives the system to explore a region in phase space far from its stable state, before returning to it. This behavior is manifested in the laser in the form of a spiking behavior, where sudden intensity dropouts happen followed by slow recoveries of its intensity.

Another relevant system where excitable behavior has been reported are neurons^26^, and a lot of research is being done to understand excitability in both systems, and to use semiconductor lasers with feedback to mimic biological neurons^27–30^.

This spiking behavior of the laser is consequence of the interplay between nonlinear light-matter interactions, time delay from feedback, and spontaneous emission noise^31^. This behavior takes place for low to moderate optical feedback and around the emission threshold of the laser. As we increase the pump current of the laser the LFF dynamics yields to coherence collapse, where the oscillations are too fast and irregular, and the dropouts cannot be distinguished.

Here we study the complex dynamics of the output intensity of a semiconductor laser with optical feedback, in the spiking regime of LFFs. We find that, i) at the onset of the LFF regime, and at the transition from the LFF regime to the coherence collapse regime, the dynamics is characterized by two competing behaviors that can be identified with a thresholding method; and ii) in these transition regimes, temporal correlations in the global spiking dynamics can be used to forecast transitions between dynamics, i.e., when the system is performing one type of spike and changes to perform another type of spike. For the range of parameters where the system performs well developed LFFs, where all the dropouts have about the same depth, there is only one type of event and forecasting does not make sense.

## Results

**Figure 1:**
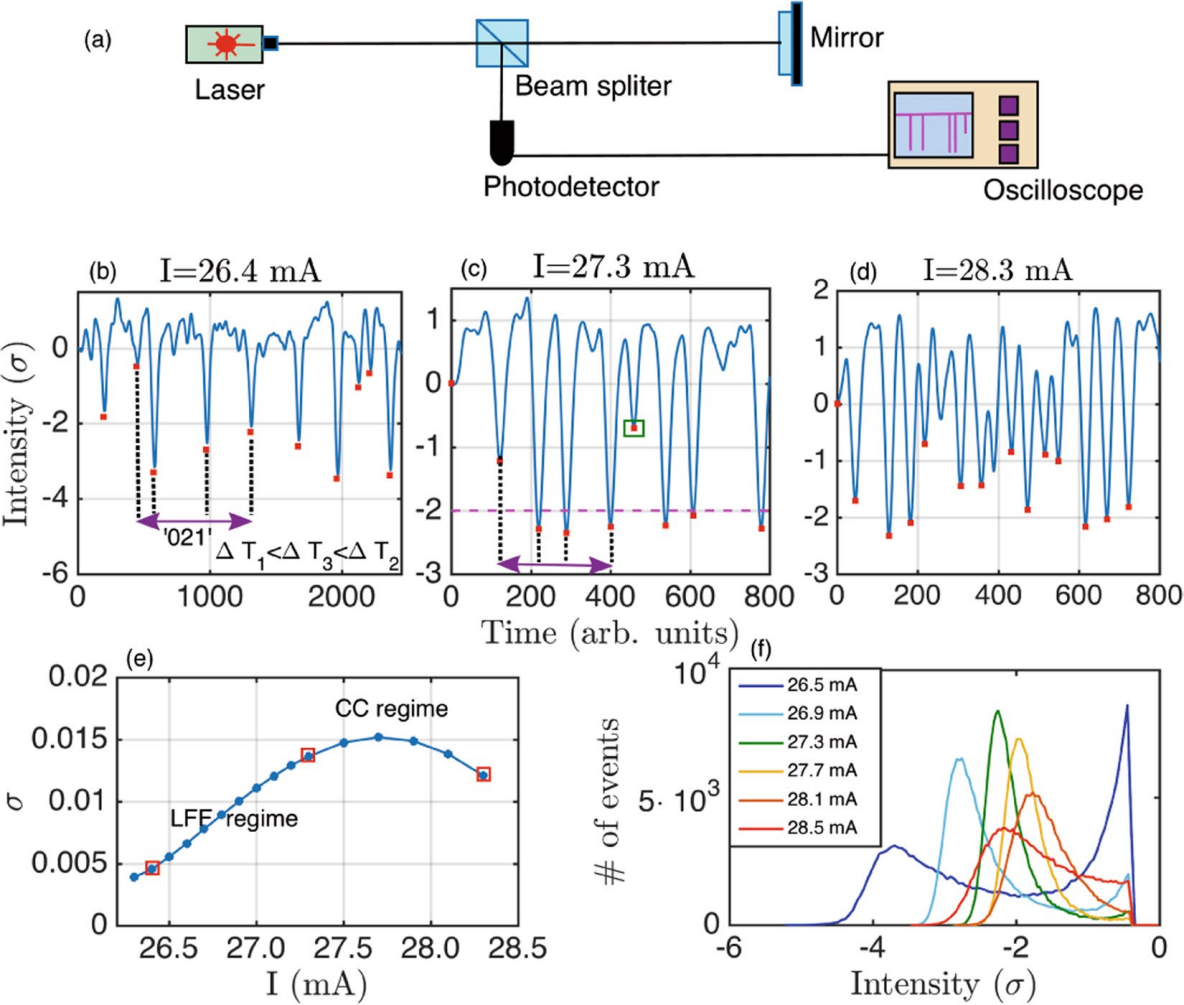
**(a)** Experimental setup. **(b)** Time series of the output intensity of the laser for *I* = 26.4 *mA*, onset of the LFFs. Red squares indicate the detection of the dropouts. One word is shown as example, ‘021’, considering the time intervals of the events (dropouts). There is a broad distribution in depths of the dips. **(c)** Time series for *I* = 27.3 *mA*, well developed LFFs. The dropouts are less spread-out. One word (double arrow) that forecasts the change from deep events to a shallow event (green square) is shown as example (see main text). Threshold is set at *th* = −2*σ*. **(d)** Time series for *I* = 28.3 *mA*, coherence collapse. **(e)** Standard deviation of the time series versus pump current. The uniform increase in *σ* corresponds to the region of LFFs. The change in trend indicates where coherence collapse (CC) begins. Red squares corresponds to the pump currents of the time series of (b), (c), and (d). **(f)** Histograms of the depths of the dropouts for various pump currents. As pump current increases the distribution goes from two-mode spread-out to single mode narrow to broad.

Figure 1b,c and d show typical time series of the semiconductor laser with feedback in the LFF regime for three values of the pump current. Because the intensity range of the time series changes with the pump current, for a correct comparison, time series have been normalized to have zero mean and unit variance. The dropout events are indicated with red squares. In our study we scan the pump currents in the regime where the LFF dynamics is present (from 26.3 mA to 28.7 mA). We increase the pump current of the laser from the onset of the LFFs, for low pump currents, through well developed LFFs, and to coherence collapse, at higher pump currents. See Methods and ref.^32^ for details of the experimental setup. Figure 1e shows the standard deviation, *σ*, of the time series. The LFF regime is characterized by a uniform increase of *σ* with the pump current. Transitions in the dynamics are characterized by a change in the trend of *σ*^33^.

To study the spiking dynamics of the system we use the ordinal patterns analysis introduced by Bandt and Pompe^34^. This analysis method transforms a time series of *N* events into *N*−*D* ordinal patterns of dimension *D*, also referred to as words. These words are computed by comparing consecutive inter-event time-intervals, *TI*(*i*) = *t*(*i*) − *t*(*i* − 1), where *TI* stands for time interval, and *t*(*i*) is the time where event *i* occurs. The number of different words, *D*!, depends on the dimension of the words, *D*. For dimension *D* = 2 we have two words: ‘01’ for *TI*(*i*) < *TI*(*i* + 1) and ‘10’ for *TI*(*i* + 1) < *TI*(*i*). For dimension *D* = 3 we have six words: ‘012’ for *TI*(*i*) < *TI*(*i* + 1) < *TI*(*i* + 2), ‘021’ for *TI*(*i*) < *TI*(*i* + 2) < *TI*(*i* + 1), etc. Words are computed with non-overlapping intervals, even though starting the analysis in word *i*, *i* + 1, or *i* + 2 does not change the words probabilities from the time series. It could happen in this type of analysis that two or more consecutive intervals where equal. This would be indicating some regularity in the system^35^, or experimental sampling limitations. In our experimental data no ties appear in the time series. Figure 1b depicts one word as example. This method has been shown to be efficient unveiling time correlations in complex time series^35–40^.

A preliminary analysis of the distribution of the events (see Fig. 1f for the histograms computed with the depths of the dropouts) shows that, as we increase the pump current the distribution of the events changes shape, from a two-mode spread-out distribution to a single-mode narrow distribution, and back to a long tail distribution. This suggests that, for low and high pump currents, the dynamics might be generated by two competing behaviors, one that triggers shallow events and another that triggers deep events. These two types of events would be contributing to each of the two peaks in the bimodal distribution in Fig. 1f, for *I* = 26.5 *mA* for example. In a previous paper^41^ it was shown that the intensity dropouts of this optical system are triggered by stochastic noise and by an underlying deterministic dynamics, showing different statistical behavior.

In order to determine and distinguish the two dynamics we define an event as an intensity dip (red squares in Fig. 1b,c,d), and consider an intensity threshold to separate them into shallow (above threshold) and deep (below threshold) events. Figure 1c shows a threshold of −2*σ* as example. Once detected all the events we calculate the probabilities of the words of dimension 3 (6 possible words).

**Figure 2:**
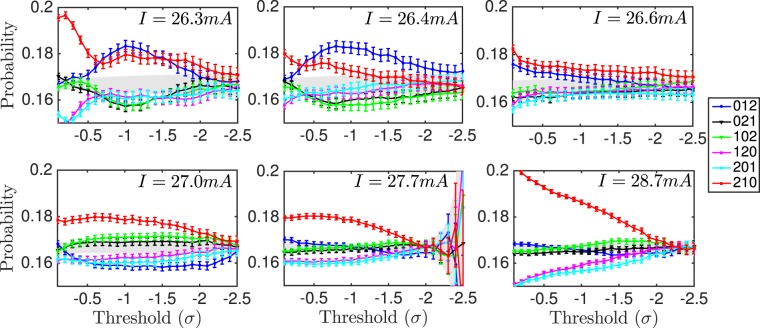
Words probabilities of dimension 3 versus threshold, computed with the time intervals between events, for different pump currents. Only events deeper than the threshold are considered. The error bars represent the confidence interval computed with a binomial test, corresponding to a confidence level of 99.7%.

Figure 2 shows the words probabilities versus threshold for different pump currents. The words are computed considering the time intervals between events (dropouts), and only events below the selected threshold are considered. The gray region corresponds to the probability values consistent with the null hypothesis that there are no temporal correlations in the sequence of dropouts, and all the words are equally probable (*p* ± 3*σ*_*p*_, where *p* = 1/*D*! and ${\sigma }_{p}=\sqrt{p\mathrm{(1}-p)/N}$, being *N* the number of words in the sequence).

For low pump currents (onset of the LFFs) the hierarchy of the words shows a dependence on the chosen threshold. This points to the ability of the thresholding method to resolve different aspects of the dynamics. There is a transition as we lower the detection threshold and exclude shallower events. The temporal correlations when shallow events are considered are different to the ones with only deep events, pointing to a different dynamics when only deep events are considered, versus when deep and shallow events are considered.

For intermediate pump currents (well developed LFFs) the hierarchy of the words does not change with threshold, and the probabilities are closer to the gray region. Remember that in this region the distribution of depths of the dropouts is narrow (see Fig. 1f), suggesting one single type of events. For high pump currents the probabilities are further away from the gray region for shallow thresholds, indicating a more deterministic behavior. There is also a subtle crossover of probabilities at around *σ* = −1. In all cases, the deepest thresholds correspond to a behavior compatible with a stochastic process (probabilities within the null hypothesis region). This indicates that either all temporal correlations are lost, or this thresholding method is unable to resolve different aspects of the dynamics of the system in this regime.

**Figure 3:**
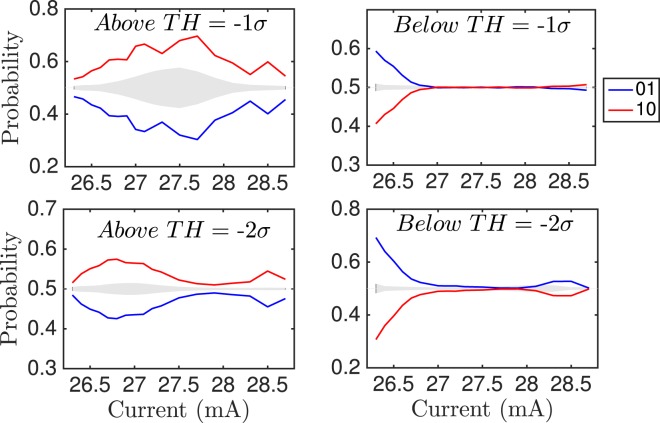
Words probabilities of dimension 2 versus current. Words are computed with the time intervals between events, considering consecutive dropouts that are either above or below the threshold. Top corresponds to *th* = −*σ*, bottom corresponds to *th* = −2*σ*. Left row corresponds to dropouts above threshold. Right row corresponds to dropouts below threshold.

To confirm the robustness of the dual behavior for shallow and deep dropouts, we compute the words probabilities of the time intervals with those consecutive events that are above the threshold, and below the threshold. Also, to filter noise out, we use an upper threshold at −0.3*σ*, and discard events whose dips are higher than this threshold. Figure 3 shows the words probabilities of dimension 2 for two different thresholds (*th* = −*σ*, and *th* = −2*σ*). We can see that the hierarchies and the behavior are different for words of dropouts above and below *th*. For dropouts below the threshold the dynamics shows strong temporal correlations for low pump currents, being the word ‘01’ more probable than ‘10’. For higher currents the behavior is clearly compatible with a stochastic process. For dropouts above the threshold, the dynamics shows clear temporal correlations for the whole pump current range, being the word ‘10’ more probable than ‘01’, the opposite than below threshold.

In many physical systems it is valuable to be able to predict changes in the dynamics, and to forecast extreme events in their dynamics, where the definition of extreme event depends on each particular system^16–18,20,42^. In our optical system, because the global dynamics can be seen as the result of the interaction and competition of two behaviors, that can be separated by a threshold, it is interesting to be able to forecast when each type of event, deep or shallow, will happen.

**Figure 4:**
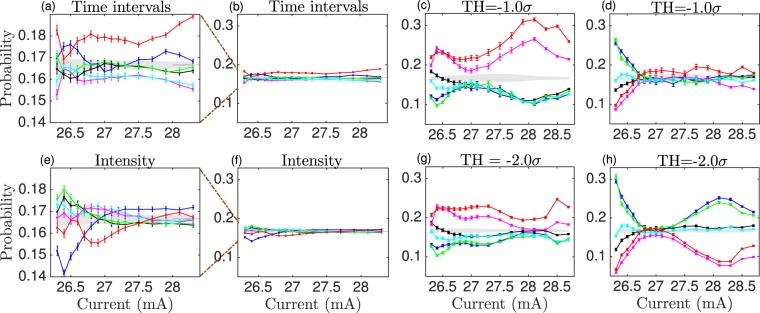
**(a)** Words probabilities computed with the time intervals between all the dropouts of the time series. **(e)** Words probabilities computed with the depths of all the dropouts of the time series. **(c**,**d**,**g**,**h)** Words probabilities that forecast the change in dynamics. **(c**,**g)** Forecast from a shallow event to a deep event. **(d**,**h)** Forecast from a deep event to a shallow event. Two different detection thresholds are shown, −1.0*σ* (**c**,**d**), and −2.0*σ* (**g**,**h**). **(b**,**f)** are the same plots as **(a**,**e)** but with the same vertical scale as the forecast plots. The probabilities of the forecast plots are considerably larger than those with the raw dropouts.

Figure 4c,d,g,h show the probabilities of words of dimension 3 that forecast a change in the dynamics. Figure 4c,g correspond to the probabilities of the words that take place before the change shallow-to-deep, i.e., the word that occurs before a deep event, as far as the previous one is a shallow event. 4 d and 4 h correspond to the probabilities of the words that take place before the change deep-to-shallow (see Fig. 1c for an example).

In order to forecast the deeper events, but also the change from one type of dynamics to the other (from shallow events to deep event and vice versa), we calculate the probabilities of the words that happen right before a deep event, being the previous event a shallow event. We define deep and shallow events as those lower and higher than the threshold, respectively. We also calculate the probabilities of the words that happen right before a shallow event, being the previous a deep event. Figure 1c shows as example one word that forecasts the change from dip-events dynamics to a shallow event.

One has to be careful when using ordinal patterns to forecast events when the range of values is finite, as some bias might be introduced, i.e., after a very deep event the probability of a higher event is high, or after a very short time interval the probability of a larger time interval is high. That is not the case in our analysis, as we compute the words with time intervals, while the threshold is in the intensity (see Fig. 1c).

## Discussion

Figure 4 shows that clear temporal correlations are present before the system goes from one type of dynamics to another (deep to shallow or shallow to deep) for low and high pump currents, which corresponds to the regimes where the system shows two coexisting dynamics. Here, the system tends to perform some preferred words before it goes from shallow to deep (‘120’ and ‘210’) and from deep to shallow (‘012’ and ‘102’). The ability to forecast depends clearly on the choice of threshold, *th*. A suitable threshold will separate better the two types of events, while a poor choice of threshold will classify events wrongly. For the shallow-to-deep prediction, a threshold between *th* = −0.5*σ* and *th* = −1.0*σ* assures that the events above *th* are shallow events and no deep events are wrongly classified as shallow. For the deep-to-shallow prediction, *th* = −2.0*σ* assures that the events below *th* are deep events and no shallow events are considered as deep.

In the central range of pump currents, where the LFFs are well developed, and their depths are similar, the forecast is not possible, all six words are equally probable, not showing strong temporal correlations that allow us to make any prediction.

It is also worth to compare the values of the probabilities for the forecasting plots (Fig. 4c,d,g,h) with those where the words are computed without imposing any forecasting condition. Figure 4b,f show the probabilities of the raw dropouts in the same vertical scale as those of the forecasting plots for comparison purposes. Even though there is an underlying structure in the dynamics of the raw dropouts, as can be seen in Fig. 4a,e, and also in ref.^32^, the magnitude of that behavior is not comparable with the one presented in the shallow-to-deep and deep-to-shallow transitions. The range of the probabilities for the raw dropouts is between 0.14 and 0.19, while the range for the prediction analysis is between 0.05 and 0.30. This difference is more relevant for low and high pump currents, which happen to be the transition regimes to, and from the LLFs.

**Figure 5:**
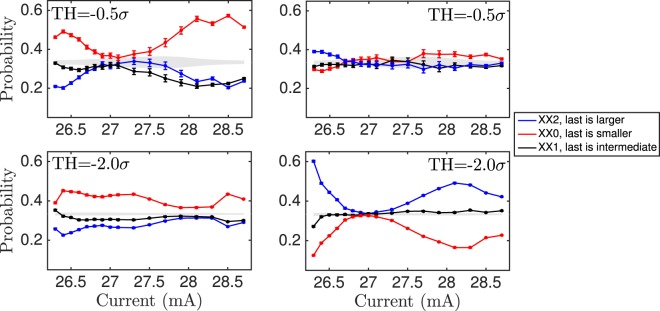
Probabilities of the forecasting time intervals combinations *XX*2 = 012 + 102, *XX*1 = 021 + 201, and *XX*0 = 210 + 120. These combinations highlight the fact that, independently of the two preceding time intervals, the last one tends to be larger (*XX*2) or shorter (*XX*0) before the transition in the dual-dynamics regime. These temporal correlations are lost in the well-developed-LFFs regime. Left row corresponds to shallow-to-deep transition. Right row corresponds to deep-to-shallow transition.

Because the preferred words and less preferred words before a change in dynamics are those for which the last time interval is larger or shorter than the preceding two time intervals, it is helpful to plot the probabilities of the combinations of words that show this global behavior. Figure 5 shows the probabilities of these combined words (‘XX2 = 012 + 102’, ‘XX0 = 120 + 210’, ‘XX1 = 201 + 021’).

It is in the dual-dynamics regimes where the system allows to forecast changes in the dynamics. There, the temporal correlations present before the deep-to-shallow transition are opposed to those before the shallow-to-deep transition. For some pump currents, more than 50% of the transitions occur after the same combination of time intervals, ‘XX0’ for shallow-to-deep (Fig. 5a), while the combination ‘XX2’ before a transition is of the order 20%. For deep-to-shallow transitions, the combination ‘XX2’ is higher than 50% for the extreme currents (Fig. 5d), while the probability of transition after the combination ‘XX0’ can be less than 20% for those pump currents.

It is worth mentioning here that the temporal correlations in the deep events, before a deep-to-shallow transition, is present even though those events are further apart as the threshold is reduced, in opposition to the loss of correlation that could be expected, indicating a strong temporal correlation.

The decrease in the predictability for the highest pump currents indicates that the transition from LFF to coherence collapse (CC) is leading to a dominant CC regime. This indicates that the method only allows to predict transitions in a dual-dynamics regime.

The words computed with the raw dropouts (Fig. 4b,f) have more similar probabilities (closer to 1/6) than those computed in the forecasting conditions (Fig. 4c,d,g,h). This indicates that, even though the system presents clear different preferences when transitioning from shallow-to-deep and deep-to-shallow, these differences are washed out when considered all the events to construct the words. Also, the fact that the system presents preferred words before the transitions suggests that the system could be exploring a specific region in phase space when these transitions are induced. As the preferred words are different for the two types of transitions, this could indicate that the region that triggers one of the transitions is different from the one that triggers the other transition.

Other analysis can be performed by considering longer embedding delay, in order to explore longer temporal correlations. This consists in computing the words by skipping a determined number, *n*, of events, i.e., (*i*, *i* + *n*, *i* + 2*n*). We computed the words with different embedding delays but no clear temporal correlations were detected beyond *n* = 1.

To summarize, we have used an ordinal patterns analysis to analyze the complex dynamics of the output intensity of a semiconductor laser with feedback. We have uncovered that in the transition regimes to, and from the spiking LFF regime (low and high pump currents respectively), its complex dynamics is the result of the competition of two different behaviors. These two behaviors trigger shallow and deep events, respectively. For the dual-dynamics regimes, we have found strong temporal correlations preceding the transitions from shallow to deep events and vice versa. This allows to forecast when the following event will be of a different type than the previous one. The temporal correlations that precede a change in the type of events is opposed if it is from shallow to deep or the other way around.

These results may be applied to other spiking complex systems that present dual-dynamics, in order to distinguish them and predict the occurrence of each one of them. Particularly, this can help current efforts in high performance information processing, such as optical neurons or neural networks.

## Methods

The experiment was performed with a 675 nm Al-GaInP semiconductor laser (AlGaInP Sanyo DL-2038-023) with optical feedback from a diffraction grating (see Fig. 1a). The external cavity length was 70 cm, with a feedback delay time of 4.7 ns. A beam-splitter was used to send 50% of the light to a 1 GHz oscilloscope (Agilent Infiniium 9000), and 50% of the light to the reflecting mirror to induce feedback to the laser. The laser temperature and pump current were controlled with a ITC502 Thorlabs laser diode combinator controller, to an accuracy of 0.01 C and 0.01 mA respectively. The operating temperature was 18 C and the laser pump current was varied in steps of 0.1 mA, from 26.3 mA to 27.3 mA, and in steps of 0.2 mA from 27.5 mA to 28.7 mA. At 18 C the threshold current of the solitary laser is *I*_*th*_ = 27.8 *mA*, and the feedback-induced threshold reduction is 6.5%. We recorded time series of 32 ms for each pump current, containing between 70,000 and 300,000 spike events, for low and high pump current respectively. A complementary analysis of the data can be found in ref.^32^.
